# Bidirectional roles of neutrophil extracellular traps in oral microbiota carcinogenesis: A systematic review

**DOI:** 10.1016/j.tranon.2025.102361

**Published:** 2025-04-15

**Authors:** Jie Shen, Haitao Lin, Kangnan Mo, Zhong Liang, Yan Zhang, Huatao Quan, Xing Wang, Chenping Zhang, Chao Chen

**Affiliations:** a Department of Head and Neck Surgery, Zhejiang Cancer Hospital, Hangzhou Institute of Medicine (HIM), Chinese Academy of Sciences, Hangzhou, Zhejiang 310022, China; b Postgraduate training base Alliance of Wenzhou Medical University (Zhejiang Cancer Hospital), Hangzhou, Zhejiang, 310022, China; c College of Stomatology, Shanghai Jiao Tong University School of Medicine, Shanghai 200233, China

**Keywords:** Neutrophil extracellular traps, Oral microbiota, Oral squamous cell carcinoma, Carcinogenesis

## Abstract

**Background:**

Neutrophil extracellular traps (NETs) are network structures composed of DNA, histones, and antimicrobial proteins,released by activated neutrophils to trap and eliminate extracellular pathogens. Recent research has demonstrated a strong correlation between NETs and various diseases, including immune dysregulation, thrombosis, and malignancies. This review synthesizes current research on NETs, focusing on its biological role in oral squamous cell carcinoma (OSCC) and explores its potential in treating.

**Methods:**

A literature review in the PubMed database was conducted to examine the impact of NETs on the homeostasis of oral microbiota and the involvement in the development of oral microbiota-related carcinogenesis.

**Results:**

Various microorganisms, including *Porphyromonas gingivalis, Fusobacterium nucleatum, Streptococcus* spp., along with *Candida albicans*, as well as certain viruses such as Human papillomavirus (HPV), Human herpes virus 8 (HHV-8), and Herpes simplex virus-1 (HSV-1)are regulated by NETs during oral colonization and proliferation and have been identified as contributors to the pathogenesis of oral squamous cell carcinoma. NETs have been shown to play a dual role in the carcinogenic process of oral microbiota in humans. At the initial stage of tumor formation, NETs inhibit tumorigenesis by eliminating tumorigenic bacteria that infiltrated the tumor; however, following tumor establishment, various cytokines and chemokines that promote tumor progression are released by neutrophils during the NETs formation.

**Conclusion:**

This article reviews the oncogenic mechanisms of NETs in the oral microbiota, with potential implications for early tumor detection and the development of microbe-targeted therapies.

## Research highlights

• Elucidate the role of oral microorganisms in the development of oral squamous cell carcinoma.
• Systematically elucidated the bidirectional role of neutrophil extracellular traps in oral microbiota carcinogenesis.
• Explored the potential value of NETs as a biomarker in precancerous screening and prognostic diagnosis of oral squamous cell carcinoma.

## Graphical abstract

**Figure:**
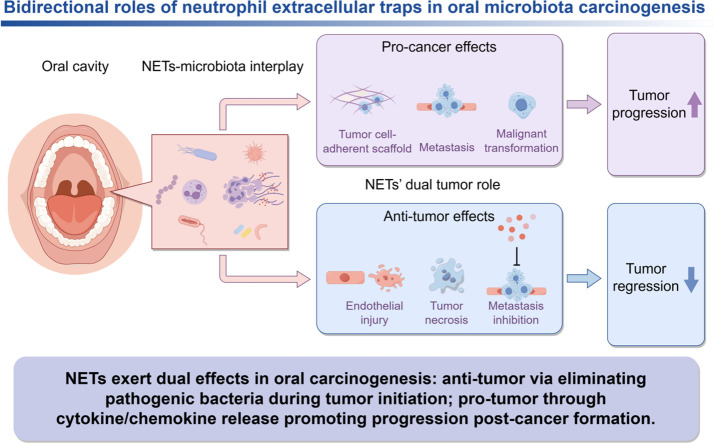
The dual role of NETs in oral carcinogenesis.

## Introduction

Neutrophils, as members of the leukocyte family, are the most abundant circulating leukocytes. They are the primary responders to acute inflammation, swiftly arriving at their targeted locations. Additionally, research conducted in recent decades has highlighted their significant involvement in chronic inflammation [1]. The mechanisms of anti-inflammatory effects involve direct phagocytosis of pathogens, production of reactive oxygen species (ROS), degranulation, release of neutrophil extracellular traps (NETs) for capturing microorganisms, expression of class II major histocompatibility complexes, and antigen presentation [2].

In 2004, Brinkmann et al. [3] identified an extracellular scaffold structure outside neutrophils known as NETs, which ensnare and eliminate bacteria. Composed of DNA and histones, NETs serve as a vital innate immune defense mechanism [4]. Neutrophils migrate to the site of inflammation and form NETs to entrap invading pathogens, exposing the pathogens to toxic proteins before ultimately being cleared by phagocytosis [5]. Nevertheless, the deleterious effects of these poisonous properties on the host can manifest when neutrophils release NETs uncontrollably in the context of persistent inflammation. Consequently, the accumulation of NETs may result in the occlusion of blood vessels, tissue damage, and chronic inflammation, thereby contributing to the advancement and exacerbation of various pathological conditions [6]. Specifically, the excessive release of NETs have been implicated in the exacerbation of autoimmune diseases and infectious diseases, including systemic lupus erythematosus, type I diabetes, COVID-19, and anti-neutrophil cytoplasmic antibodies-related vasculitis [[7], [8], [9], [10]].

NETs released within the tumor microenvironment have been found to facilitate the aggressive advancement of various cancers, such as glioblastoma, pancreatic cancer, breast cancer, and OSCC [11]. However, accumulating evidence suggests that the role of NETs in inhibiting tumor occurrence and development cannot be ignored. Schedel et al. [12] showed that neutrophil-induced NETs exert cytotoxic effects on melanoma cells in co-culture systems, leading to tumor cell necrosis. The current studies have reported the role of the NETs in the cancer process, but the results are completely different, thus pointing out the functional duality of the NETs.

The oral microbiota ranked as the second largest microbiota in the human body following the intestinal microbiota, plays a significant role in shaping the intricate tumor microbial microenvironment in oral squamous cell carcinoma [13]. Dysregulation of the oral microbiota has been implicated in inducing inflammation within the oral cavity and is closely associated with the development and advancement of oral cancer. Additionally, NETs are particularly important in maintaining the homeostasis of the oral microbiota. Currently, no systematic research addresses the binary role of NETs in the malignant transformation of oral microbiota. This article mainly focuses on the potential carcinogenic role of NETs in the oral microbiota.

## Methods

### Eligibility criteria

Studies were included if they involved human subjects, human-derived samples (e.g., oral tissues, saliva, blood), or cells, and focused on the mechanisms, biomarkers, or NETs and OSCC or oral potentially malignant disorders (OPMD). Exclusion criteria comprised: (1) studies solely addressing non-oral cancers or non-microbial carcinogenic mechanisms; (2) low-quality reviews, editorial letters, case reports, or conference abstracts; (3) duplicate publications; (4) non-English language publications; (5) animal experiments or in vitro studies lacking clinical relevance.

### Study selection and data extraction

Two independent reviewers conducted initial screening of titles and abstracts using standardized forms for data extraction. Full-text reviews determined final inclusion. Extracted data included: first author, publication year, study country, case/control sample sizes, tumor characteristics (anatomical location/TNM stage), control group type (healthy/benign/OPMD), sample type (blood/saliva/tissue), and key findings linking NETs and oral microbiota to carcinogenesis.

### Quality assessment

Observational studies were evaluated using the Newcastle-Ottawa Scale (NOS), categorized into high-, medium-, or low-quality tiers based on scores. Discrepancies were resolved via discussion or consultation with a third reviewer. To comprehensively analyze NETs in oral cancer, this study integrated original research and high-quality reviews to reveal field-wide consensus and discrepancies. The AMSTAR 2 tool assessed review quality, retaining only high.

## Results

### Article selection

**Fig. 1:**
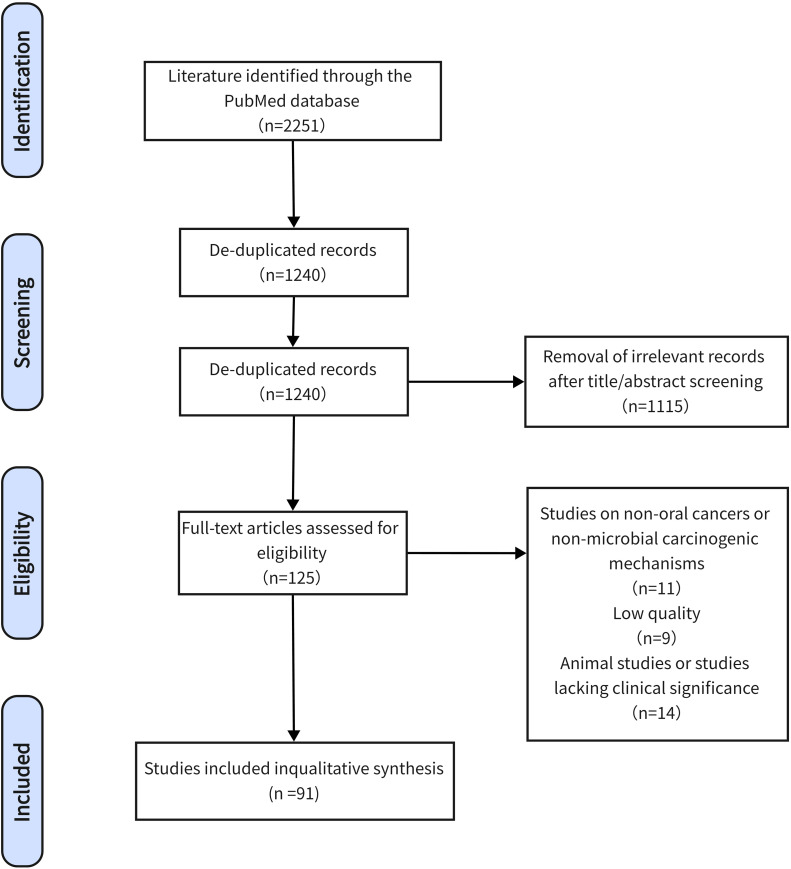
Flow chart based on PRISMA guidelines.

The explore search and selection strategies as shown in the flow chart in Fig. 1. A preliminary literature search identified 2251 articles that met the preliminary search criteria. After screening, 91 articles were included in the descriptive systematic review.

### The structure and function of NETs

The skeleton of NETs is composed of chromatin filaments measuring approximately 15–17 nm in diameter, encased by spherical structures measuring approximately 50 nm in diameter, consisting of primary and secondary neutrophil granules, resulting in a distinctive ultrafine network configuration [14,15]. Chromatin filaments consist of nuclear DNA or mtDNA and histones, with histone core consisting of H2A, H2B, H3, and H4, accounting for 70 % of the total trap proteins [16]. Globular structures within NETs primarily contain neutrophil elastase (NE), antimicrobial peptides, myeloperoxidase (MPO), gasdermin D (GSDMD), leukocyte protease 3, tissue protease G, granule proteins, α defensins, and calgranulin, etc. [3,17]. Histones in NETs primarily package DNA into chromatin, possessing potent antibacterial properties and cytotoxic effects. During nuclear envelope collapse and chromatin disintegration, nuclear envelope components (NE) from secretory granules bind to dispersed chromatin and migrate to the extracellular matrix, accumulating at infection sites. Cyclin-dependent kinases 4/6 (CDK4/6) and Protein kinase C (PKC) then induce nuclear envelope rupture, allowing NE to enter the nucleus. This process ultimately degrades cytotoxic factors and eliminates bacteria [18]. Conversely, MPO gains access to the phagocytic cells through passive diffusion via nuclear pores, thereby eliminating engulfed microorganisms within the cell [19]. GSDMD's main function after activation is to drill holes on the granular membrane to promote NE release [17].

### The release and clearance mechanisms of NETs

The process by which neutrophils release NET is called NETosis, which is another way of cell death in addition to apoptosis and necrosis. The generation methods of NETs include suicidal and non-suicidal NETosis, with the latter further categorized into cell nucleus DNA release-dependent types that do not require ROS participation and mitochondrial DNA (mtDNA) release-dependent types that require ROS participation [20]. Suicidal NETosis is activated by microbial products, platelets, pro-inflammatory cytokines or other signals, and subsequently relies on large amounts of ROS, PKC, and Raf-MEK-ERK signaling pathways produced by NADPH oxidase (NOX), as well as histone citrullination mediated by type IV peptidylarginine deiminase (PAD4), ultimately leading to chromatin depolymerization, nuclear membrane rupture and release of NETs containing chromatin and cytoplasmic proteins, followed by neutrophil death [14,[20], [21], [22]]. In contrast, non-suicidal NETosis, also known as vital NETosis, does not require activation of the Raf-MEK-ERK signalling pathway. In non-suicidal NETosis, neutrophils bind Toll-like receptor (TLR) or complement receptor to C3 protein-ligand to change the morphology of the nuclear membrane [23,24]. This results in the release of NETs through vesicle budding, followed by the re-fusion of the cell membrane to maintain its original functionality, allowing for continued chemotaxis and phagocytosis [24].

Although the formation mechanisms of NETs have been extensively investigated, their degradation and clearance mechanisms remain poorly understood, with this imbalance potentially constituting a critical juncture in disease progression. Current evidence indicates NETs degradation primarily depends on DNA-degrading enzymes and macrophage-mediated clearance, where the enzymatic process requires coordinated actions of DNase family members (DNase I, DNase II, DNase1L3) and TREX family nucleases (TREX1, TREX2): DNase I cleaves double-stranded DNA to eliminate intravascular NETs [25], DNase II degrades exogenous DNA in lysosomal acidic environments [25,26], while DNase1L3 secreted by macrophages synergizes with DNase I for NETs clearance [25,27,28]; concurrently, TREX1 degrades oxidized DNA and modulates the cGAS-STING pathway (deficiency causing autoimmunity and genomic instability) [25,[29], [30], [31]], with TREX2 participating in DNA repair and mRNA transport [25,30,32]. Macrophages phagocytose NETs via macropinocytosis/lysosomal pathways, exhibiting enhanced efficiency through DNase pretreatment and pro-inflammatory polarization (LPS+IFN-γ) [27,28], whereas dysregulated NETs degradation may trigger inflammation, autoimmunity, and tumor progression [25,27,28,33].

### Oral microorganisms and NETs

Normal homeostasis between the oral microbiota and the human host is essential for maintaining normal body functions [13]. Disruption of this balance can contribute to the development of various oral diseases such as gingivitis, periodontitis, dental caries, pulpitis, and even squamous cell carcinoma of the oral cavity [13,34]. As an open digestive organ rich in various microorganisms, the oral cavity receives approximately 30,000 oral polymorphonuclear leukocytes (OPMN) per minute through gingival crevicular fluid in healthy humans. Moonen et al. [35] have reported that OPMN produces 13 times more NETs compared to circulatory polymorphonuclear leukocytes (CPMN). Meanwhile, due to the particularity of the oral environment, OPMN has higher adhesion and phagocytosis to various microorganisms than CPMN. Therefore, NETs released by OPMN play a crucial role in maintaining the homeostasis of oral microbiota.

**Fig. 2:**
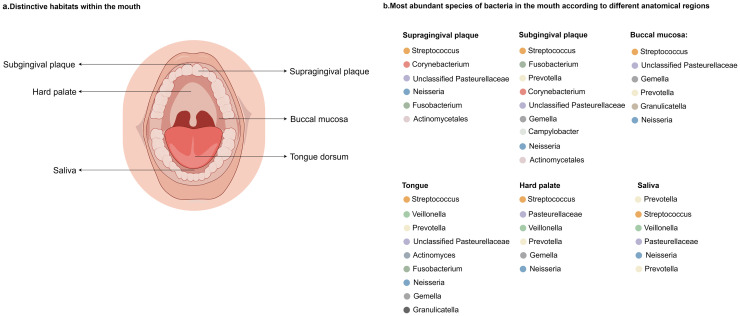
a, Microenvironments in the oral cavity suitable for the survival of different microorganisms. b, Most abundant bacterial species in different anatomical parts of the oral cavity.

Current research indicates that the human oral microbiota exhibits significant diversity, including bacteria, microeukaryotes, archaea, and viruses, which may exist as microorganisms, symbionts, and pathogens. In addition, there is a large number of uncultured ultra-small bacteria in the oral cavity, known as the "Candidate Portal of Radiation (CPR)" group [36]. Research on the oral microbiome has revealed that the human oral cavity harbours over 700 bacterial species in dozens of genera from seven phyla, including Actinomycetota, Bacteroidota, Firmicutes, Fusobacteriota, Proteobacteria, Saccharibacteria, and Spirochaetota [37]. The Human Oral Microbiome Database (HOMD) identifies Firmicutes, Bacteroidetes, Proteobacteria, Actinobacteria, and Fusobacteria as the most prevalent phyla in the adult oral cavity, with Streptococcus, Haemophilus, Leptotrichia, Porphyromonas, and Prevotella as the predominant genera [38]. Prior research has indicated that various physiological structures within the oral cavity, including the hard surfaces of enamel both above and below the gingival line, keratinized surfaces like the palate, gingiva, and lingual papillae, as well as soft surfaces such as the buccal mucosa, create microenvironments conducive to the colonization of different oral microorganisms [39]. The colonization of bacteria in the human oral cavity is shown in Fig. 2.

**Fig. 3:**
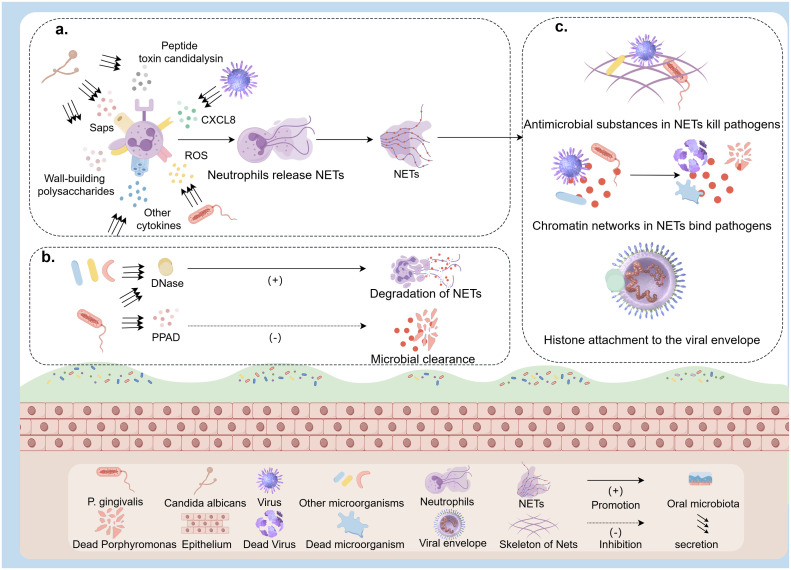
a, the process in which various microorganisms in the oral cavity secrete cytokines to promote the release of NETs by neutrophils; b, microorganisms degrade NETs by releasing substances such as DNase and PPAD, antagonizing their phagocytic activity; c, The process of NETs binding to pathogens through their network skeleton and histones, and then killing pathogens through antibacterial substances on the skeleton.

When persistent oral inflammation such as periodontitis occurs, certain components secreted by oral pathogenic bacteria (e.g., Porphyromonas gingivalis and Aggregatibacter actinomycetemcomitans) can stimulate oral epithelial cells to produce a cascade of pro-inflammatory cytokines and chemokines, promoting the formation of neutrophil extracellular traps (NETs), as illustrated in Fig. 3a. Jayaprakash et al. [40] co-cultured the Porphyromonas gingivalis (P. gingivalis) cell line ATCC33277 with neutrophils, resulting in significant secretion of CXCL8 and reactive oxygen species (ROS), further promoting the formation of NETs. The subsequent neutralization of P. gingivalis by NETs suggests a potential role for NETs in mitigating the progression of P. gingivalis-mediated infections. Stobernack et al. [41] demonstrated that the oral pathogen P. gingivalis impaired the neutralizing capacity of NETs by secreting the virulence factor PPAD, possibly through citrullination of histone H3, though the precise mechanism remains unclear, thereby facilitating bacterial colonization and the progression of periodontitis (Fig. 3b). Additionally, research suggests that NET release may exacerbate tissue damage rather than provide protection, and the release of host-derived DNasemay play a crucial role in digesting and clearing NETs in tissues [42,43]. Coincidentally, a separate study conducted a culture of 34 different types of periodontal bacteria on Luria-Bertani (LB) medium, specifically focusing on analyzing granular bacteria and extracellular DNase production from the LB cultures. The study identified 27 different types of bacteria, including P. gingivalis, Tannerella forsythia, Fusobacterium nucleatum, Parvimonas micra, Prevotella intermedia, Streptococcus constellatus, Campylobacter rectus, and Prevotella nigrescens. These bacteria expressed abundant extracellular DNase to degrade NETs (Fig. 3b), thereby blocking their neutralization and facilitating oral colonization [44]. Nevertheless, once tumor cells complete malignant transformation, P. gingivalis promotes the progression of OSCC by stimulating the release of NETs in the tumor immune microenvironment [45].

Oral virus-induced NETs generally capture and clear viruses through various direct or indirect mechanisms (Fig. 3c). The network, like the chromatin skeleton in NETs, can bind and immobilize virus particles to prevent virus spread [46]. Antibacterial substances on the chromatin skeleton of NETs, such as MPO, antimicrobial peptides, α-defensins, and other components can exert antiviral effects [42,47]. The histones present in NETs hold a high concentration of positively charged amino acids, allowing for attachment to the virus's negatively charged viral envelope and subsequent inactivation [48]. Furthermore, the constituents of NETs can indirectly eliminate viruses by triggering and enhancing the antiviral responses of other immune cells [49]. In addition, a significant proportion of viruses found in the oral cavity are bacteriophages, with data indicating that over 90 % of individuals are persistently infected with viruses from the Herpesviridae families [50]. Notably, the members of the Herpesviridae families can encode proteins with similar DNase activity and package them in the capsid, thereby degrading NETs to evade their viral-killing effect [51]. This mechanism is related to the development of human periodontal disease and OSCC [52].

**Fig. 4:**
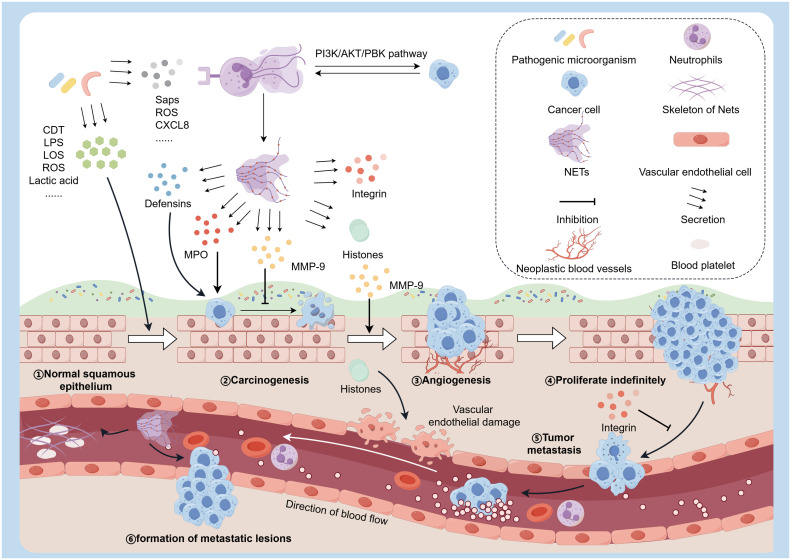
NETs promote tumor development at every step of the metastatic cascade, including early adhesion, proliferation, invasion, and angiogenesis.

Using amplicon technology, scientists have identified more than 100 genera of fungi in the oral cavity [53]. Oral fungal communities typically comprise Candida spp. or Malassezia spp. [54]. Of these, Candida albicans are believed to be linked to various oral health issues such as dental caries, periodontitis, and OSCC [55]. NETs were crucial in combating infections and neoplasms caused by Candida albicans. Candida albicans, a dimorphic fungus, can transition from single spore to mycelium cells. The peptide toxin candida lysin secreted after mycelium formation can induce the formation of NETs, phagocytose *Candida albicans*, and effectively inhibit its growth [56] (Fig. 3a). In addition, the cell wall-building polysaccharides (mannans and β-glucans) and the secreted aspartate protease (Saps), also trigger NETs at different intensities [57] (Fig. 4). The interaction between Sap4 and Sap6 secreted by hyphae and CD11b receptors activates the release of NETs (Fig. 4). Nevertheless, neutrophils release NETs through ROS-dependent mechanisms following interaction with cell wall-bound Sap9 and Sap10, utilizing CD16 and CD18 receptors for protease recognition (Fig. 4). Besides, β- Glucan triggers ROS-dependent NET production by binding to Dectin-1, CD11b, and CD18 receptors. Mannans are recognized by TLRs, CD14, and Dectin-1 receptors, mainly triggering NET release through ROS-independent pathways. A study compared the ability of two strains of Candida albicans, SC5314 and 3683, to evade NETs-mediated killing. The results showed that degrading NETs with exogenous DNase or catalase significantly reduced neutrophil killing ability in the co-culture system. Cytochalasin also suppressed neutrophil phagocytic activity [58]. Amoebas (Entamoeba gingivalis) and mitochondrial-free flagellates are less well-studied, and their main living place is in periodontal pockets, which are associated with periodontal disease [59]. NETs released by human neutrophils covered the entamoeba and reduced their vitality during the in vitro interaction. The histones, MPO, and depolymerizing chromatin secreted by NETs eliminate parasites [60] (Fig. 3c). Recent research indicates that the activation of human neutrophils through the synergistic action of interferon-γ and TNF-α leads to the release of NETs, resulting in a 70 % clearance rate of entamoeba infection [60].

### Oral precancerous lesions and NETs

Periodontitis is a relatively common oral disease, with some clinical signs of periodontal disease present in 50–70 % of adults. Periodontitis is believed to initiate from a disruption in bacterial ecology [[61], [62], [63]], with current studies identifying pathogens such as Aggregatibacter actinomycetemcomitans, P. gingivalis, Tannerella lithia, Treponema denticola, Prevotella intermedia, Prevotella nigrescens, Par-vimanas micra, Campylobacter rectus, and Fusobacterium nucleatum, suggesting that ecological imbalances and the proliferation of pathogenic species may play a significant role in the onset of periodontitis. Patients with periodontitis have a 2–5 times higher risk of developing many types of cancer than healthy individuals, especially OSCC [61,64,65]. The heightened risk may be attributed to changes in the oral microbiota composition, specifically involving key pathogenic organisms such as P. gingivalis, Prevotella intermedia, and Fusobacterium nucleatum [64,[66], [67], [68]]. These microorganisms are implicated in inducing genetic modifications in epithelial cells through various mechanisms [[69], [70], [71]], including inflammation, cell survival promotion, activation of oncogenic pathways, release of virulence factors, and facilitation of cellular transformation, consequently, this cascade of events can stimulate abnormal cellular proliferation, potentially culminating in the initiation and progression of cancer. NETs can be detected in the purulent exudate of periodontal pockets, gingival crevicular fluid, and plaque biofilms containing neutrophils in patients with periodontitis [72,73]. The accumulation of significant levels of NETs within the periodontal pockets of individuals afflicted with periodontitis has been documented as effectively eliminating pathogenic bacteria, including P. gingivalis [40,74]. This observation supports the role of NETs in the immune defense of periodontal tissues by facilitating the eradication of pathogenic microorganisms. Specifically, neutrophils are recruited to the site of periodontal inflammation to release NETs, thereby contributing to the mitigation of inflammation, containment of oral pathogens, and the progression from chronic gingivitis to OSCC.

Oral potential malignant disorder (OPMD) is a collection of oral mucosal lesions collectively characterised by a range of morphological changes, some of which may have increased potential for malignant transformation. These precancerous lesions such as oral leukoplakia (OLK), oral erythroplakia (OEK), and palatal lesions in smokers, as well as precancerous conditions like oral submucous fibrosis (OSF), actinic keratosis, oral lichen planus (OLP), and discoid lupus erythematosus (DLE). However, not all OPMD diseases will transform into cancer [38,75]. OLP, OLK, and OEK are more common in the human oral cavity and have a higher risk of malignant transformation [75].

OLK, the most common form of OPMD, consists mainly of white plaques of uncertain risk, with a 9.7 % likelihood of malignant transformation reported in the literature [76]. In an early study, patients with OLK had an increased abundance of Clostridium spp. and a decreased abundance of Bacteroides spp. as well as more prevalent Candida colonization and a highly variable pattern of bacterial colonization [77]. Based on the data analyzed, it was determined that oral microorganisms exhibit variations in individuals with OLK compared to those in good health. However, further research is needed to explore the potential synergistic or antagonistic impact of NETs and oral microbiota on the progression of OLK towards malignancy. Among the various subtypes of OMPD, OEK has an intermediate risk of malignant transformation, perhaps related to its lower prevalence and thus few studies have observed the underlying changes in bacterial microbiota in patients with OEK [38,78]. Oral lichen planus (OLP) is a common oral mucosal disease characterized by chronic inflammation, with a malignancy rate of approximately 0.1–2 %. The etiology and pathogenesis of OLP are still not elucidated, preliminary clues have demonstrated that various causes may be involved, including infection, impure oral hygiene, autoimmunity, dysregulation of oral microbial ecology, and stress. Analysis of the oral microbiome has revealed an ecological dysregulation of the microbiome in patients with OLP, mainly characterized by low levels of fungi and higher levels of bacteria [79]. OLP patients differed from healthy individuals in oral microbiology and microbiomes of tissues and saliva. Capnocytophaga and Gemella were higher in the saliva of OLP patients, while Escherichi-Shigella and Megaspaera were higher in the tissues of OLP patients. Additionally, seven taxa including Carnobacteriaceae, Flavobacteriaceae, and Megaspaera were enriched in both the saliva and tissues of OLP patients. The aforementioned flora exhibiting increased abundance has been demonstrated to be correlated, to varying degrees, with abnormal immune reactions and the production of inflammatory responses. Jablonska et al. [80] isolated neutrophils from the peripheral blood of patients with OLP displaying heightened ability to form NETs, and the excessive NETs formation induced by TGF-β in OLP may potentially contribute to the transformation into OSCC.

### Oral microbiota, oral squamous cell carcinomas and NETs

The oral microbiota of patients with OSCC exhibits significant alterations in composition and abundance compared to precancerous and healthy individuals. As previously mentioned, the most abundant phyla in healthy, precancerous, and OSCC patients are Fusobacteriota, Bacteroidota, Proteobacteria, and Firmicutes. Some microorganisms are overrepresented in OSCC, such as Capnocytophaga, Fusobacterium, Leptotrichia, Neisseria, Bergeyella, Mycoplasma, Johnsonella, and Staphylococcus. Fusobacteriota constituting 34 % of the bacterium detected in OSCC patients [81,82].

The current limitation is that most studies focused on revealing changes in the microbiomes of OSCC and healthy individuals, and it is difficult to say whether these microbial alterations are the cause or the consequence of the disease. It is currently established that some certain microorganisms, such as bacteria (P. gingivalis, Fusobacterium nucleatum, Streptococcus sp., Capnocytophaga sp., and Oral Campylobacter), viruses (HPV, HHV-8, HSV-1, and Epstein-Barr Virus), and Candida albicans, are associated with the etiology and advancement of OSCC [82]. As previously discussed in this paper, NETs produced by oral neutrophils can impede the colonization and progression of various bacteria, fungi, and viruses, thereby potentially hindering the malignant transformation of oral microorganisms leading to OSCC. Once the completion of malignant transformation, NETs participate in the biological development of tumors in various ways different from intervening in microbial infections. Studies have demonstrated the presence of NETs in OSCC. Marzena Garley et al. compared NETs-related markers in tissues, peripheral blood, and saliva between healthy individuals and OSCC patients, revealing that MPO and histones were detected in tumor tissues of 6 out of 16 cases (37.5 %), with concurrent low expression of NOX in tumors. Interestingly, lower concentrations of NOX1, Neutrophil Cytosolic Factor 2 (NCF2), and DNase were observed in saliva, whereas elevated levels were detected in serum. The authors speculate that this phenomenon might be associated with compensatory activation of peripheral blood neutrophils to offset the deficiency of NETs-related proteins in saliva. These dynamic changes warrant further investigation for their potential applications in diagnosis and treatment efficacy monitoring [83].

The effect of NETs on tumor inhibition or promotion is still unclear. Schedel et al. [12] found the anti-tumor effects of NETs in vitro. Specifically, NETs adhere to melanoma cells through integrin-mediated adhesion, inhibiting migration (Fig. 4). In addition, MPO on NETs showed cytotoxicity to melanoma cells, causing necrosis. Histones, another key component of NETs, were shown to cause damage to endothelial cells and subsequently disrupt the nourishing blood vessels of tumors [84] (Fig. 4). Additionally, defensins within the neutrophil extracellular trap were observed to lyse tumor cells. The impact of NETs on tumor growth is influenced by the specific tumor type and microenvironment, with recent research indicating a greater promotion of tumor development by NETs (Fig. 4). NETs play a role in various stages of the metastatic process, such as adhesion, proliferation, invasion, and angiogenesis. The matrix metalloproteinase 9 (MMP-9) in NETs can inhibit tumor cell apoptosis, facilitating angiogenesis and tumour neovascularisation [85] (Fig. 4). Simultaneously, NETs can serve as scaffolds for platelet adhesion, activation, and aggregation to promote thrombosis [86,87]. Li et al. [88] found that NETs enhanced procoagulant activity in patients with OSCC. Additionally, NETs can augment the adherence of cancer cells to the endothelial lining and potentially facilitate metastasis by enhancing tumor cell extravasation [89]. Table 1 summarizes the mechanisms and biological functions of NETs and their components in both pro-tumorigenic and anti-tumorigenic roles. Recent studies have shown that in the co-culture system of OSCC cell lines and neutrophils, cancer cells regulate the PI3K/Akt/PBK pathway to interact with neutrophils, resulting in increased release of NETs, thereby promoting tumor cell growth [90].Current evidence indicates that *Porphyromonas gingivalis* (P. gingivalis) promotes the progression of oral squamous cell carcinoma (OSCC) by stimulating neutrophil extracellular traps (NETs) release within the tumor immune microenvironment [91]. The specific oncogenic mechanism may be associated with NETs-mediated secretion of matrix metalloproteinase-9 (MMP9) and various cytokines.

**Table 1:** The mechanisms and biological functions of NETs and their components in both pro-tumorigenic and anti-tumorigenic roles.

Effect Type	Key Substances	Mechanism	Biological Effect
Pro-carcinogenic	MMP-9	Inhibits tumor cell apoptosis	Promotes tumor survival, angiogenesis, invasion, and metastasis
	Cytokines	Activates inflammatory and pro-carcinogenic signaling pathways	Drives chronic inflammation, tumor proliferation, and immune evasion
	NETs scaffold	Acts as a physical scaffold for platelet and tumor cell adhesion	Promotes thrombogenesis and tumor cell metastasis.
Anti-carcinogenic	Histones	Damages endothelial cells	Disrupts tumor vasculature
	Defensins	Lyses tumor cell membranes	Induces tumor cell necrosis or apoptosis
	MPO	Exhibit cytotoxicity against melanoma cells	Induction of necrosis in tumor cells
	Integrins	NETs bind tumor cells via integrins (e.g., in melanoma)	Suppresses tumor cell migration

### The potential value of NETs as a biomarker

Most patients are in the middle or late stage when they are diagnosed because malignant tumors are usually asymptomatic in the early stage of development, presenting challenges in treatment. Early detection, diagnosis, and treatment can significantly improve the prognosis, increase the survival rate, and improve the quality of life. The various cancer-regulating mechanisms of NETs, coupled with the properties of neutrophils as the most abundant immune cells and the first responders to inflammation, suggest their potential as a serum marker for early screening and prognostic prediction of tumors. The potential value of NETs as a predictor has been show in Table 2. Circulating levels of NET-related components have been evaluated as screening or prognostic biomarkers in gastric, breast, ovarian, and renal cancers [93,[102], [103], [104]]. Elevated circulating levels of NETs are thought to be associated with poor prognosis in cancers such as B-cell lymphoma, colorectal cancer and hepatocellular carcinoma [[105], [106], [107]]. In esophageal cancer, it was shown to be an independent prognostic factor [108]. Zhang et al. [103] found that NETs had a higher diagnostic value than carcinoembryonic antigen 199 in gastric cancer by comparing the ROC curves of patients with gastric cancer. In a research investigation of hepatocellular carcinoma (HCC), the authors developed the NETs Formation Potential Score model to forecast the prognosis of HCC patients and evaluate the effectiveness of immunotherapy, demonstrating favorable performance in validation across multiple cohorts [96].

**Table 2:** The potential value of NETs as a predictor in various diseases.

Author	Predictive factors	Cancer	Predictive efficiency
Zhan et al. [92] .	Circulatory NETs	HBV-related HCC	AUC for Progression:0.70 (95 % CI, 0.62–0.78) AUC for metastatic: 0.83 (95 % CI, 0.75–0.91)
Qu et al. [93].	NETs-associated genes	Gastric Cancer	AUC for prognosis: 1 years: 0.602; 2 years:0.634; 3 years: 0.692
Jin et al. [94].	Immunohistochemical staining for NETs (CitH3 +)	Pancreatic Ductal Adenocarcinoma	C-index for prognosis: NETs vs TNM^8^th: 0.6193 vs 0.6994
Jiang et al. [95].	NETs-related lncRNAs	Breast cancer	AUC for prognosis: 1 years: 0.779; 2 years:0.715; 3 years: 0.700
Xin et al. [96].	NETs-associated genes	Hepatocellular carcinoma	AUC for prognosis: 1 years: 0.836; 2 years: 0.879; 3 years: 0.902
Lin et al. [97].	NETs score model (ACSS2, MCAM, PPM1M, CYP2S1)	Osteosarcoma	AUC for prognosis: 1 years: 0.798; 2 years: 0.792; 3 years: 0.804
Jia et al. [98].	Circulating NETs	Adult-onset Still's disease	AUC for patients who were refractory to low-dose glucocorticoid: 0.917
Gao et al. [99].	circulating free-DNA/NETs	sepsis	AUC for sepsis patients with liver injury: 0.932
Ibrahim et al. [100].	serum NETs	diabetic foot ulcer	AUC for diabetic foot ulcer: 0.727
Chen et al. [101].	Risk model constructed by six NET-related genes.	head and neck squamous cell carcinoma	AUC for Progression: 3 years: 0.743; 5 years: 0.743

In studies investigating NETs as biomarkers for OSCC, ROC curves of NET-related markers (MPO and H3cit) demonstrated their significant predictive capability for clinical staging, survival status, and TN staging in OSCC patients, with both serving as independent prognostic factors [91]. Elevated NETs levels were strongly associated with poorer patient outcomes, advanced pathological staging, larger tumor size, lymph node metastasis, aggressive invasion patterns, recurrence risk, and high neutrophil-to-lymphocyte ratios [91,109]. However, an earlier 2018 study revealed increased NETs production by neutrophils in stage I/II OSCC patients, while stage III/IV OSCC showed NETs levels comparable to controls [110]. This discrepancy may be attributed to the selection of detection markers and highlights the limitations of relying on a single NETs indicator for prognostic prediction. To address this, Chen et al. ^101^developed a novel predictive model for head and neck squamous cell carcinoma that evaluates six NET-related genes to improve prognostic accuracy. Nevertheless, the model remains constrained by the incomplete understanding of NET-related gene functions and their interactive mechanisms.

Although many studies have reported the potential value of NETs as serum markers for early tumor screening or prognostic prediction, many problems are still waiting to be solved, for instance, challenges persist in accurately detecting key components of NETs, such as circulating free DNA (cfDNA), nucleosomes, citrullinated histone H3 (citH3), and NET-related proteins like NE and MPO. The development of standardized NETs assays with enhanced specificity is crucial to address these limitations. Neutrophil degranulation can also produce NE and MPO without necessarily forming NETs, and cfDNA can also be produced by apoptotic and necrotic cells. Therefore, the diagnostic specificity of NE, MPO and cfDNA for the formation of NETs is not satisfactory. CitH3, a product of PAD4-mediated citrullination, is currently regarded as the most specific biomarker for the detection of NETs during their formation [102,111]. Another problem is that NETs can be detected in various tumors, and thus may lack specificity for diagnosing a particular cancer type. Consequently, using NETs in conjunction with traditional tumor markers is necessary for accurate diagnosis and prognostication. Many related literatures are also limited by sample size and various factors with biased results, and there is an urgent need to expand the samples for multicenter studies to help its early application in the clinic.

Current research on neutrophil extracellular traps (NETs) as biomarkers in oral squamous cell carcinoma (OSCC) remains limited, though existing studies consistently highlight their significant research potential. Future investigations should prioritize three directions. First, combining multiple indicators or identifying key biomarkers to screen representative markers of NETs levels, thereby developing standardized and highly specific detection methods. Second, expanding sample sizes, refining subgroup classifications (e.g., by clinical stage or pathological features), and conducting clinical validation to clarify specific predictive applications rather than broadly associating NETs with adverse outcomes, pathological types, or TNM staging. Additionally, mature biomarkers require establishing detailed diagnostic criteria and comparative validation against traditional clinical markers. Third, improving OSCC-specific diagnostic specificity is essential, as NETs are detectable across multiple cancer types. Leveraging OSCC's unique characteristics—such as superficial lesion accessibility, the feasibility of saliva-based biomarker detection, and its close association with microbial communities—may provide critical insights for future research.

## Conclusion

NETs play an important role in maintaining the homeostasis of oral microbiota, deeply participating in the malignant transformation of oral microbiota and the malignant progression of OSCC. In the early stage of tumor formation, NETs inhibit tumor development by killing oncogenic bacteria infiltrating the tumor; However, once a tumor forms, NETs release various cytokines and chemokines to promote tumor progression. Therefore, targeted NETs therapy has certain application prospects in OSCC; The differential abundance of NETs—higher in serum and lower in saliva—may serve as a biomarker for OSCC.

## Ethics approval and consent to participate

Not applicable.

## Consent for publication

Not applicable

## Availability of data and materials

All data of this research is included in this published article.

## CRediT authorship contribution statement

**Jie Shen:** Writing – review & editing, Writing – original draft, Visualization, Validation, Methodology, Formal analysis, Data curation, Conceptualization. **Haitao Lin:** Writing – original draft, Visualization, Data curation, Conceptualization. **Kangnan Mo:** Writing – original draft, Methodology, Data curation. **Zhong Liang:** Formal analysis, Data curation. **Yan Zhang:** Validation, Investigation, Conceptualization. **Huatao Quan:** Formal analysis, Data curation. **Xing Wang:** Investigation, Conceptualization. **Chenping Zhang:** Writing – review & editing, Supervision, Project administration, Data curation, Conceptualization. **Chao Chen:** Writing – review & editing, Supervision, Project administration, Investigation, Data curation.

## Declaration of competing interest

The authors declare that they have no competing interests.
